# Daniel McNaughton (1813-1865)

**DOI:** 10.4103/0019-5545.37328

**Published:** 2007

**Authors:** T. V. Asokan

**Affiliations:** Department of Psychiatry, Institute of Mental Health, Chennai, India

Daniel McNaughton was the son of a Glasgow wood turner. He was harboring a delusion that there was a conspiracy against him, and he perceived harassment by the spies sent by Catholic priests with the help of Jesuits and Tories.

The following was the revelation by McNaughton during interrogation in Bowstreet police station. “The Tories in my native city have compelled me to do this. They followed me to France, into Scotland and all over to England. In fact, they follow me wherever I go… They have accused me of crimes of which I am not guilty; they do everything in their power to harass and persecute me. In fact they wish to murder me.”

The Commissioner of Police was aware of McNaughton's condition for 18 months before the shooting incident. Two years before the shooting incident, McNaughton asked him to put a full stop to the persecution and later reminded him to apply to the Sheriff. His delusions were directed against the Tories, and he decided to kill the Tory Prime Minister Sir Robert Peel.

On 20^th^ June 1843 Edward Drummond, the Private Secretary of Sir Robert Peel, was coming out of the Prime Minister's residence and McNaughton mistook him for Peel. He followed him out of Whiteall Garden and in Parliament Street; and in front of numerous spectators, he shot him in the back and he died five days later.

McNaughton was arrested by a constable who had witnessed the incident and was taken to BowStreet police station. At the inquest in BowStreet, the verdict was willful murder and McNaughton was indicted.

Dr. Edward Thomas Monro in Bethlem Hospital examined McNaughton for the defense at NewGate prison. Sir Alexander Morrison, Dr. A. J. Sutherland (Physician, Sir Luke's Hospital) and Mr. William McClure (Harley Street Surgeon) were present during the examination of McNaughton. During subsequent examinations by Monro, Dr. Hutcheson (Physician to Royal Asylum) and Dr. Crawford (Glasgow) were present. Mr. Aston Key of Guy's Hospital; and Dr. Philips, Surgeon and Lecturer at the West Minister's Hospital, had examined McNaughton and gave evidence at the trial.

Dr. Forbes Benignus Winslow, an authority in insanity defense, was called. (He did not examine McNaughton and remained as a spectator throughout the trial - a controversy - and House of Lords questioned him about this.)

During the trial, Alexander Cockburn (counsel for defense), asked Dr. Monro whether the delusions of McNaughton were real or assumed. Dr. Monro confirmed that the delusions were real and considered that the killing was committed under a delusion and McNaughton carried out of an idea which had haunted him for years. All others who gave evidence confirmed that McNaughton was insane. Alexander Cockburn had made extensive and excessive use of Isaac Ray's Treatise on the Medical Jurisprudence of Insanity. Cockburn quoted extensively from the book, which rejected traditional views of the insanity defense based on the defendant's ability to distinguish right from wrong in favor of causation. When Dr. Forbes Benignus Winslow and Dr. Philips (both appeared for the Crown) concurred with the opinions of other doctors called by defense, the case collapsed. Terms like homicidal monomania and partial delusion were discussed during the trial, and the foreman jury without the retiring jury returned a verdict of insanity.

McNaughton was acquitted of murder; and considering insanity, he was forcibly institutionalized for the rest of his life under Criminal Lunatics Act 1800. He was first remanded to Bethlem Royal Hospital (stayed there for 20 years); and in 1864 he was transferred to Bradmoor Asylum, and he died on 3^rd^ May 1865 at the age of 52. The establishment and the press protested the verdict. Queen Victoria was displeased to a greater extent and wrote to Sir Robert Peel for a wider interpretation of the verdict.

On 6^th^ March 1843, there was a discussion in the House of Lords, and Lord Chancellor put five questions to a panel of His Majesty's judges. The five questions were replied on 19^th^ June 1843, and they were construed as McNaughton's rules. The following are the main points of McNaughton's rules:

1. Every man is to be presumed to be sane and to possess a sufficient degree of reason to be responsible for his crimes, until the contrary be proved.
2. An insane person is punishable “if he knows” at the time of crime.
3. To establish a defense on insanity, the accused, by defect of reason or disease of mind, is not in a position to know the nature and consequences.
4. The insane person must be considered in the same situation as to responsibility as if the facts with respect to which the delusion exists were real.
5. It was the jury's role to decide whether the defendant was insane.

McNaughton's rules stressed on “understandability of right and wrong” and “intellectual” rather than a moral or affective definition dominated in its formulation. Lack of control and irresistible drives or impulses were neglected. In essence, it was the “test of knowing” or “test of right and wrong.” If McNaughton's rules had been applied to McNaughton at the time of trial, he could not have been found guilty on the grounds of insanity.

The spellings for his name vary in many instances, but hospital records and court documents support the spelling “McNaughton”.

Section 84 IPC embodies McNaughton rules as follows: “Nothing is an offence which is done by a person who, at the time of doing it, by reason of unsoundness of mind, is incapable of knowing the nature of the act or that he is doing what is either wrong or contrary to the law.”
